# Vaccine hesitancy in patients presenting to a specialized allergy center: clinical relevant sensitizations, impact on mental health and vaccination rates

**DOI:** 10.3389/fimmu.2024.1324987

**Published:** 2024-05-17

**Authors:** Natalie Kogseder, Viktoria Puxkandl, Wolfram Hoetzenecker, Sabine Altrichter

**Affiliations:** ^1^ Department for Dermatology and Venereology, Kepler University Hospital, Linz, Austria; ^2^ Center for Medical Research, Johannes Kepler University, Linz, Austria; ^3^ Institute of Allergology, Charité – Universitätsmedizin Berlin, Corporate Member of Freie Universität Berlin, Humboldt-Universität zu Berlin, and Berlin Institute of Health, Berlin, Germany; ^4^ Allergology and Immunology, Fraunhofer Institute for Translational Medicine and Pharmacology (ITMP), Berlin, Germany

**Keywords:** allergy, COVID, vaccine, prick-test, Hospital Anxiety and Depression Scale (HADS)

## Abstract

**Introduction:**

The COVID vaccination program with new types of vaccinations and early reports of allergic reactions to vaccines led to vaccination hesitancy in patients with allergies. In this study, we aimed to characterize patients who present at an allergy center with specific questions regarding risk assessment to COVID vaccines in comparison to regular allergy center patients.

**Methods:**

A total of 50 patient charts of patients with risk assessment for COVID vaccination (COV group) and 50 regular allergy center patients (ALL group) were assessed for documented allergies, comorbidities, total IgE, and tryptase levels and hospital anxiety and depression score (HADS). Skin prick testing (SPT) with additives of COVID vaccines [polyethylene glycol (PEG), polysorbate] were performed if indicated based on medical history.

**Results:**

Patients who presented for examination prior to a possible COVID vaccination were mostly female (86%) and had more frequently reported allergic reactions to drugs in the past, but only in a minor group (28%) were the reactions qualified as anaphylaxis. The group COV patients scored significantly higher in the HADS for anxiety and depression than the regular group ALL patients. The same trend was observed when data were corrected for gender. It is worth noting that patients without any prior contact to COVID vaccines scored comparable regarding anxiety to patients with prior reaction to COVID vaccinations, but significantly higher in the depression score. In 19 patients (38%) who met the indications for SPT for the suspicious contents PEG and Polysorbate 80, the tests did not show a positive result. Furthermore, 84% of patients underwent the prick test, but only 15% of patients who received consultation alone agreed to vaccination at our center. No vaccination-related event was documented in these patients.

**Discussion:**

In conclusion, vaccination hesitancy was frequently elicited by negative experiences with drugs and putative drug allergies. Female patients predominate in this patient group, and the anxiety and depression scores were significantly elevated. Allergological workup, including SPT, led to a high rate of subsequent vaccinations, whereas a discussion with the patients about risks and individualized advice for vaccination without testing only rarely resulted in documented vaccinations.

## Graphical Abstract

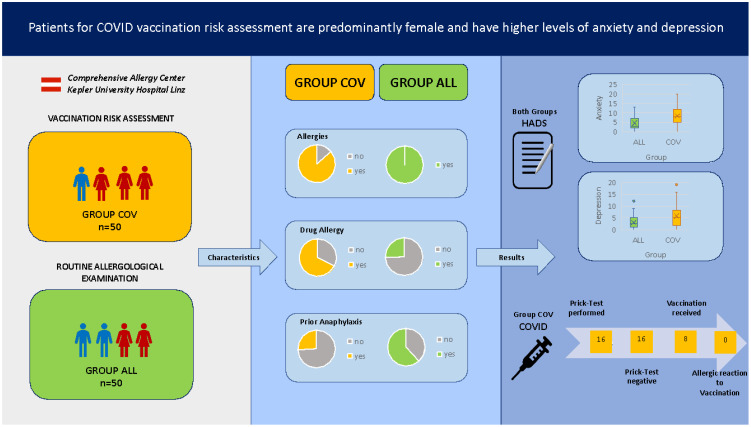

## Introduction

Since 2019, the emerging coronavirus SARS-CoV-2 pandemic has affected people all around the globe, psychologically as well as physically. During pandemics in general, higher levels of anxiety appear to be a huge problem (1). Besides anxiety, depression is also described to be more prevalent during a pandemic (2). In particular, people with pre-existing anxiety disorders are confronted with higher stress levels in pandemic situations compared to people without a mental illness (3). Besides anxiety being associated with the pandemic itself, the administration of new vaccines that have also been suspected to trigger intolerance reactions can provoke uncertainty and anxiety—which may both result in vaccine hesitancy (4).

Vaccinations, in general, rarely result in life-threatening anaphylactic reactions, with an estimated rate of 1.3 in one million people. In this context, it has been observed that the vast majority (85%) reacting to vaccines were diagnosed with a concomitant atopic disease (5). At the very beginning of the COVID-19 vaccination program in the United Kingdom (UK) in December 2020, the report on two anaphylactic reactions associated with the COVID-19 vaccines resulted in warnings regarding these new substances. The Medicines and Healthcare Products Regulatory Agency in the UK, on this matter, recommended at that time to exclude from COVID vaccination the patients with any anaphylactic reaction to food, drugs, or vaccine—which resulted in an initial vaccination hesitancy in patients with allergy-related background (6). After later reevaluation, the European Academy of Allergy and Clinical Immunology (EAACI) announced that only patients with proven allergies against vaccination components meet the absolute contraindications (7). In mRNA vaccines (BioNTech/Pfizer and Moderna), polyethylengylcol (PEG)-2000 is the suspected ingredient to cause allergic reactions (8). PEG is a polymer, which can vary in size with a maximum of about 5,000 g/mol. In general, PEGs are often part of several drugs, e.g., penicillin, various laxatives, or injectable corticosteroids, as a stabilizer for lipid nanoparticles. A small number of type-I IgE-mediated allergic reactions against PEG had been described, especially when administering big amounts of substances containing PEG (9). Vaccines based on vectors (AstraZeneca and Janssen) do not contain PEG2000 but Polysorbate 80, which is equally suspected to be the culprit for potential allergic reactions upon vaccination (10).

In general, patients with allergies more often show anxiety and higher stress vulnerability (11). It has been observed that allergies are associated with psychological dysfunction, depression, anxiety, post-traumatic stress disorder (PTSD), and less stress-coping abilities during the COVID-19 pandemic. This increased occurrence is especially observed in female patients (12). Wang et al. (13) reported that patients with allergic rhinitis have 1.4- and 1.7-times-higher anxiety rate and depression score, respectively, in comparison to patients without allergies.

During the COVID-19 pandemic, the Austrian government announced an obligation for COVID-19 vaccinations. As a result, an increased number of patients presented at allergy centers to test for a possible allergy to the mentioned ingredients of the different COVID-19 vaccines, to receive a waiver for vaccination, or, in case of a given risk profile, to be vaccinated against coronavirus under allergological supervision.

To our knowledge, it had not been analyzed in the past which patients sought medical advice regarding potential allergic risk upon COVID vaccination in comparison to patients who were due for routine allergological examination. Accordingly, we aimed to analyze the following: (i) what were the characteristics of patients who presented for allergological examination regarding COVID-19 vaccination and in how many patients could a potential risk for allergic reaction upon vaccination be verified? (ii) How was their mental status in comparison to those of routine allergological patients during the COVID-19 pandemic? (iii) How was the vaccination rate in these patients and what were the potentially influencing factors?

## Methods

### Patients

Between June 2021 and June 2022, patients who presented with an explicit question regarding their potential risk upon COVID vaccination were seen at the allergy outpatient clinic of the Department of Dermatology and Venereology, Comprehensive Allergy Center, Kepler University Hospital. A total of 50 patient files were randomly selected for this study (patient group COV). To serve as the control group, 50 patient files of those with allergic diseases that did not present for this specific reason (patient group ALL) were chosen. All analyses were performed retrospectively and pseudonymized.

Ethical approval was obtained from the local ethics committee (ECS no. 1152/2022). The retrospective data analysis did not require a written informed consent since all patients’ records were handled in a pseudonymized manner following data protection regulations and in agreement with local ethics. The clinical characteristics and demographics of all patients are presented in **Table 1**.

**Table 1:** Cohort characteristics.

	Patient group ALL *n* = 47	Patient group COV *n* = 50	Significance, *p*
Age (year) Median (IQR) Mean (± SD)	58.0 (27) 54.4 ± 16.8	53.5 (19) 52.3 ± 13.3 (*n* = 46)	0.139
Sex (m:f)	**26:21**	**7:39**	**<0.001**
**Mastocytosis**	8.5%	0% (*n* = 39)	0.298
**Allergies in anamnesis**	**100%**	**86.1%** (*n* = 43)	**0.01**
**Number of different allergy categories** Median (IQR) Mean (± SD)	* * *1.0 (1.0)* *1.6 ± 0.8*	* * *2.0 (2.0)* *2.0 ± 1.3 (n* = *43)*	* * *0.097*
**Prior to anaphylaxis**	**61.7%**	**28.6%** **(*n* = 42)**	**< 0.001**
**Allergic rhinitis**	29.8%	36.4% (*n* = 44)	0.598
**Asthma**	14. 9%	16. 7% (*n* = 42)	0.856
**Atopic dermatitis**	2.1%	4.8% (*n* = 42)	0.604
**Urticaria**	10.6%	9.8% (*n* = 41)	0.717

Group COV include patients with concerns about possible anaphylactic reactions toward a vaccine compound, while group ALL include the control group or regular allergy center patients. Results are shown as median ± IQR of the indicated number of individual data points or independent experiments. Mann–Whitney *U*-test was used for age and chi-square test for other assessments. *P*-values <0.05 were considered statistically significant. Significant values are displayed in bold, while trends (*p* <0.1) are in italic.

y, years; SD, standard deviation; IQR, interquartile range; m, male; f, female.

### Clinical assessments

Patients’ data, including age, sex, documented allergies, and concomitant diseases, were obtained from the patients’ charts.

The patients’ records were screened for documented allergies. Allergies were divided into six categories: aeroallergies, food allergies, contact allergies, venom allergies, allergies against injectable drugs, and allergies against oral medication. If patients had one or more documented allergies that fitted into one category, this category would be considered “positive”.

Anaphylaxis was defined as prevalent if it has been documented that the patient had suffered in the past from immediate-type reactions with extracutaneous symptoms like dyspnea or other symptoms that can be assigned to grade 2 anaphylactic reactions according to Ring and Messmer’s (14) grading scale.

Regarding concomitant allergic diseases, the patients’ charts were screened for asthma, atopic dermatitis, allergic rhinitis, and urticaria. Mastocytosis was defined as prevalent if tryptase >11.4 μg/L and further diagnostic measures confirmed the diagnosis (15).

Clinical data were missing in three patients of group ALL and in up to 11 patients in the group COV patients.

### Laboratory assessments

Total IgE and tryptase serum levels were assessed in the central nuclear laboratory of the clinic using the ImmunoCAP System^®^ (Phadia Laboratory Systems, Thermo Fisher Scientific Inc., Uppsala, Sweden). Total IgE level >112 IU/mL and tryptase level >11.4 µg/L were considered elevated.

#### Skin prick test

If the anamneses identified any prior reaction of the patients that could be in any doubt related to possible COVID vaccine ingredients like PEG or Polysorbate 80, a prick test with PEG 400, PEG 2000, PEG 4000, and Polysorbate 80 solutions was performed. [for the detailed protocol, see (10)]. Histamine (0.1%) served as positive control and saline solution (sodium chloride 0.9%) of Company Fresenius as negative control. After 20 min, wheal development, at 1.5 mm bigger than the saline prick, was considered a positive result.

#### Vaccination

The patients were offered vaccination at the Department of Dermatology and Venerology, Comprehensive Allergy Center, Kepler University Hospital. The available vaccines were Comirnaty (BioNTech/Pfizer), Spikevax (Moderna), and Jcovden (Johnson & Johnson). The patients were observed for 30 min after vaccination and then discharged. The patients were advised to report back to the clinic any potential allergic reactions after discharge.

#### Psychological assessment

In order to assess the anxiety and depression status of our patients, we used the Hospital Anxiety and Depression Scale (HADS) with the two different subscales: HADS—Anxiety (HADS-A) and HADS—Depression (HADS-D). Each subscale consists of seven questions regarding the mental status of the patient. Per question, 0–3 points can be achieved, resulting in a maximum score of 21 per subscale. A sum score of 0–7 points is considered not to be clinically significant, 8–10 points are considered to be a doubtful score, and 11 points or more are defined as definitely clinically significant scores (16).

### Statistical analyses

Statistical analyses were performed using SPSS (Version 29.0.0.0). Normality tests (Kolmogorov–Smirnov and Shapiro–Wilk tests) could only confirm normal distribution on age and anxiety in ALL and COV groups, depression in COV group, and the number of allergy categories in COV group. Statistical analysis was performed using Mann–Whitney *U*-test or *t*-test for group comparison. Correlations were calculated by Spearman rank correlation test, and correlation coefficients are displayed as “*r*”. Binominal variables were analyzed using chi-square test or Fisher exact test for small categorical numbers (<5). A *p*-value ≤0.05 was considered to indicate statistical significance.

## Results

### Patients with hesitation to COVID vaccination are distinct to patients with common allergies

Patients who presented for examination because of possible future COVID vaccinations (group COV) had a mean age of 52 years and were mostly female (86%). The age in our control group (group ALL) of regular allergy center patients at the same time period was comparable, but there were significantly fewer female patients (45%, see **Table 1**).

**Figure 1:**
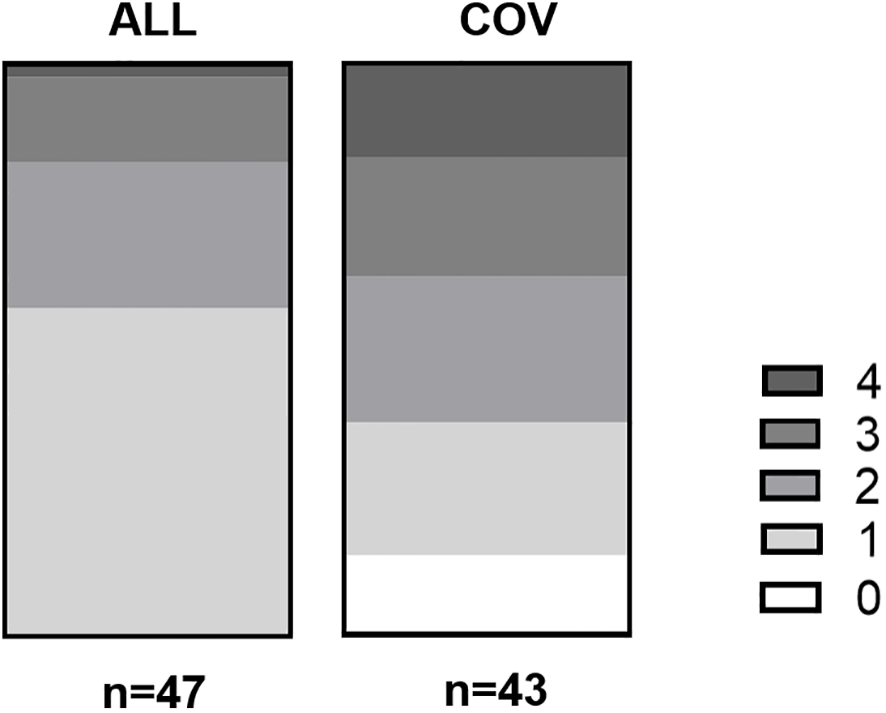
Number of different allergies form different categories in group ALL and COV. On the X-axis are the numbers of allergy per group, while the Y-axis shows the number of patients. In group ALL, 27 patients (54%) had one allergy, 12 patients (24%) had two allergies, seven patients (14%) had three allergies, and one patient (2%) had four allergies. Patient group COV, in comparison, had six patients (12%) with no allergies, 10 patients (20%) with one allergy, 11 patients (22%) with two allergies, nine patients (18%) with three allergies, and seven patients (14%) with four allergies. *p* < 0.001 in the chi-square test.

Prior anaphylaxis was documented in about a quarter of the COV patients, which was significantly lower than in the comparison group (61.7%, see **Supplementary Figure S1**). In 14% of the COV patients, no allergies could be obtained from the anamnesis or diagnostic testings. This was not the case in the ALL patient group. However, if the patients had allergies, they had significantly more often allergies that would fall into several allergy categories (see **Figure 1**).

Insect venom allergy was most prevalent in group ALL, whereas all other allergy categories showed a significantly higher prevalence in patient group COV (see **Supplementary Figures S2A, B**, **Supplementary Table S1**). Furthermore, 67.6% of group COV had any kind of anamnestic drug allergy (oral or injectable), while only 25.5% of patient group ALL had anamnestic drug allergies. Especially suspected allergies against injectable drugs were almost six times more common in group COV compared to group ALL (**Supplementary Table S1**).

Regarding asthma, atopic dermatitis, allergic rhinitis, and urticaria, there were no significant differences between the two patient groups (see **Table 1**). Diagnosed mastocytosis was rare in group ALL (8.5%) and absent in group COV (see **Table 1**).

### Patients who presented for examination regarding COVID vaccination had higher scores for depression and anxiety and could more often be clearly diagnosed with anxiety and depression compared to other patients with allergies

We used HADS questionnaires to screen the psychological status of the patients. The group of COV patients scored significantly higher for both anxiety and depression (see **Table 2**). Regarding anxiety, the highest score in group ALL was 13 points compared to 20 points in the COV group. Patient group ALL reached a maximum of 9 points in the depression score, while patient group COV had up to 19 points.

**Table 2:** HADS score of groups ALL and COV.

	Patient group ALL *n* = 50	Patient group COV *n* = 50	Significance, *p*
Anxiety			
Mean ± SD Median (IQR)	**4.5 (± 3.5)** **3.0 (5.0)** **(*n* = 50)**	**8.20 (± 4.9)** **8.0 (7.0)** **(*n* = 50)**	**<0.001**
No diagnosis Doubtful diagnosis Clear diagnosis	**40 (80%)** **6 (12%)** **4 (8%)**	**23 (46%)** **10 (20%)** **17 (34%)**	**0.001**
Depression			
Mean ± SD Median (IQR)	**3.1 (± 2.6)** **2.5 (4.0)**	**5.8 (± 4.8)** **5.0 (6.5)**	**<0.001**
No diagnosis Doubtful diagnosis Clear diagnosis	**47 (94%)** **2 (4%)** **37 (2%)**	**37 (74%)** **3 (6%)** **10 (20%)**	**0.013**

Results are shown as mean ± SD and median (IQR) of the indicated subscales or the absolute frequency of no diagnosis, doubtful diagnosis, and clear diagnosis. Numbers in brackets show the corresponding percentages.

Mann–Whitney *U*-test or chi-square test was used for comparison. *P*-values <0.05 were considered statistically significant. Significant values are displayed in bold.

SD, standard deviation; IQR, interquartile range.

Anxiety was correlated with depression and showed a strong positive correlation (*r* = 0.698), which was statistically significant at *p* < 0.001.

When patients were grouped according to their score into no, doubtful, or clear diagnosis for anxiety or depression, we observed that patients in group COV reached significantly more often the score for clearly diagnosed anxiety or depression (see **Table 2**; **Supplementary Figures S3A, B**).

### Female patients scored significantly higher than male patients in anxiety, but not in depression

**Figure 2:**
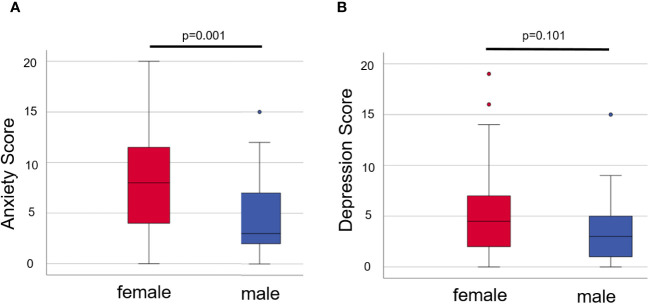
Anxiety **(A)** and depression **(B)** in female and male patients of both groups. Box plots show the mean scores from the HADS-A **(A)** or -D **(B)** with the median being characterized by the line in the box and the box showing the interquartile range. Number of females = 60, number of males = 33. Mann–Whitney *U*-test was used for comparison. *P*-values <0.05 were considered statistically significant.

On one hand, when comparing anxiety in all female and male patients, we found that female patients had significantly higher scores than male patients (*p* = 0.001, see **Figure 2A**; **Supplementary Table S2**). On the other hand, female patients did score slightly but not significantly higher in depression compared to male patients (*p* = 0.101, see **Figure 2B**; **Supplementary Table S2**).

The female patients in group ALL likewise scored significantly higher in anxiety in comparison to the male patients in group ALL (*p* = 0.038), but not significantly higher in depression (*p* = 0.399, see **Supplementary Table S3A**). In group COV, however, the male patients scored non-significantly minimally higher for depression (*p* = 0.811) and minimally lower for anxiety (*p* = 0.811, see **Supplementary Table S3B**) compared to female patients.

### Patients who presented for examination regarding COVID vaccination tend to score higher for anxiety and depression, even when corrected for gender differences

When analyzing the female patients of both groups, we found that the female patients in group COV had a trend to score higher in anxiety (*p* = 0.078) and in depression (*p* = 0.096) compared to the female patients in group ALL (see **Table 3**). However, the male patients in group COV did not have significantly higher levels of anxiety compared to the male patients in group ALL (*p* = 0.109, see **Supplementary Table S4**). In depression, a trend toward higher scores in group COV could be recognized (*p* = 0.074, see **Supplementary Table S4**).

**Table 3:** HADS score of female patients of groups ALL and COV.

	Female patients of patient group ALL *n* = 21	Female patients of patient group COV *n* = 39	Significance, *p*
Anxiety			
Mean ± SD Median (IQR)	*6.0 (± 4.1)* *6.0 (6.5)*	*8.5 (± 4.9)* *8.0 (7.0)*	*0.078*
No diagnosis Doubtful diagnosis Clear diagnosis	13 (62%) 4 (19%) 4 (19%)	16 (41%) 9 (23%) 14 (36%)	0.269
Depression			
Mean ± SD Median (IQR)	*3.6 (± 3.0)* *3.0 (4.25)*	*5.8 (± 4.8)* *5.0 (6.0)*	*0.096*
No diagnosis Doubtful diagnosis Clear diagnosis	18 (86%) 2 (10%) 1 (5%)	29 (74%) 1 (3%) 9 (23%)	0.117

Results are shown as mean ± SD and median (IQR) of the indicated subscales. Diagnosis following the HADS are shown as absolute figure (percentage). Mann–Whitney *U*-test and chi-square test were used for comparison. *P*-values <0.05 were considered statistically significant. Significant values are displayed in bold, while trends (*p* <0.1) are in italic.

SD, standard deviation; IQR, interquartile range.

### No correlation of higher levels of anxiety and depression with age or number of positive allergy categories could be verified

As we analyzed the relation between patient age and the scores of anxiety and depression, no significant correlation could be verified—neither among all patients nor if analyzed for both groups separately (data not shown).

When we analyzed the relationship between anxiety and depression scores and the different numbers of positive allergy categories, we also found no significant correlation (data not shown).

### No differences in anxiety and depression in patients with and without prior anaphylaxis or allergic comorbidities

History of anaphylaxis was not associated with higher levels of anxiety and depression compared to patients with no history of anaphylaxis, neither in group ALL (anxiety: *p* = 0.982, depression: *p* = 0.241) nor in group COV (anxiety: *p* = 0.379, depression: *p* = 0.922) and in both groups (anxiety: *p* = 0.357, depression: *p* = 0.109).

When patients were grouped according to the prevalence of allergic comorbidities (allergic rhinitis, asthma, atopic dermatitis, urticaria, and mastocytosis), we also did not see a significant difference in the scores for anxiety or depression (data not shown).

### Anxiety and depression scores correlated weakly with total IgE, but not with tryptase levels

When looking at laboratory parameters, we did know the total IgE in 47 patients of group ALL, but only in 25 patients of group COV. Still we correlated the total IgE with the scores of anxiety and depression and could verify significant relations. The total IgE in all patients correlated weakly but significantly with anxiety (*r* = 0.260, *p* = 0.027) and also with depression (*r* = 0.242, *p* = 0.041). When analyzing the levels of total IgE for our two patient groups separately, total IgE correlated stronger with depression in group ALL (*r* = 0.479, *p* = 0.015) and with depression in group COV (*r* = 0.401, *p* = 0.047). However, total IgE neither correlated with anxiety in group ALL (*r* = 0.008, *p* = 0.955) nor with anxiety in group COV (*r* = 0.046, *p* = 0.761) when analyzed separately.

Tryptase, as a further laboratory parameter, was known in 45 patients of group ALL and 21 patients of group COV. When correlating tryptase in all our patients with the scores for anxiety, no significant correlation could be observed (*r* = -0.23, *p* = 0.063). No correlation between the scores of depression and tryptase in all patients could be verified (*r* = -0.146, *p* = 0.241) likewise either. As we analyzed the relation of anxiety and depression with tryptase in each group, no correlations could be verified either (data not shown).

### Unvaccinated patients had significantly higher scores in depression compared to patients with prior vaccination, but not in anxiety

Out of 50 patients who presented for COVID vaccination examination at our clinic, 11 patients had prior vaccination, and 10 out of 11 had reported reactions to those prior vaccinations.

When analyzing anxiety and depression in patients with no prior vaccination compared to patients with prior vaccination, we found significantly higher scores of depression (*p* = 0.002, see **Supplementary Table S5**), but not for anxiety (*p* = 0.124, see **Supplementary Table S5**). While patients with prior vaccination scored in the mean 2.3 ± 2.6 points for depression, the unvaccinated patients scored 7.0 ± 4.8 points.

### Patients who presented for examination regarding COVID vaccination were not sensitized to additives of COVID vaccines and tolerated the vaccination, if performed

Out of 50 patients who presented at our clinic regarding a potential allergy against the vaccination ingredients (group COV), 19 patients (38%) met the indications for prick testing for the suspicious contents PEG and Polysorbate 80. A prick test was then performed in 16 out of 19 patients. The other three patients refused testing or were lost to follow-up. Among these 16 tested patients, no patient had a positive prick test result. Out of 16 patients with negative test results, eight patients (50%) decided to receive vaccination at the Comprehensive Allergy Center of the clinic. The remaining patients refused vaccination at our center or were lost to follow-up.

**Figure 3:**
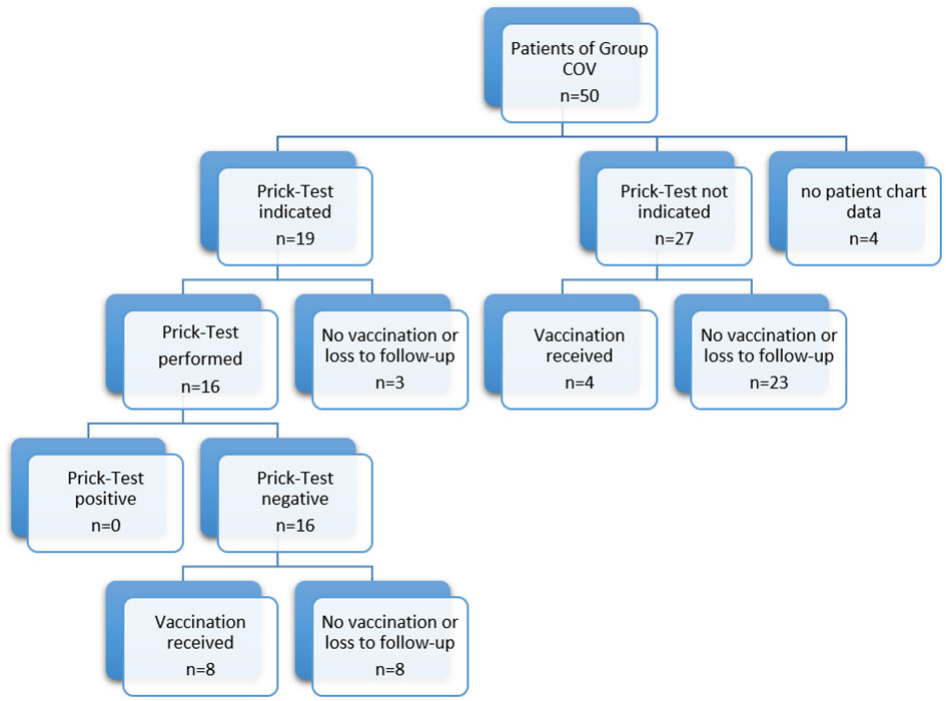
Allergological workup of the COV patient group.

A total of 27 patients of patient cohort COV did not meet any indication for prick testing. Four out of 27 patients (15%) with no anamnestic allergy risk received the vaccination at the Comprehensive Allergy Center of the clinic. The remaining patients refused vaccination at our center or were lost to follow-up (see **Figure 3**).

None of the patients reacted to the vaccination with immediate- or delayed-type allergic reactions.

### Patients who were lost to follow-up scored significantly higher in anxiety and depression compared to patients who received vaccination after the allergological risk assessment

In the course of allergological risk assessment in patients of group COV, 12 patients received COVID vaccination at our center, but 34 did not and were lost to follow-up. Patients who were lost to follow-up scored significantly higher in anxiety and depression compared to patients who got vaccinated at our center after the examination (see **Table 4**).

**Table 4:** HADS score of patients who received (with vaccination) and who did not receive a vaccination at the allergy center (without vaccination) after allergological risk assessment in group COV.

	Patients with vaccination after risk assessment *n* = 12	Patients without vaccination after risk assessment *n* = 34	Significance, p
Anxiety			
Mean ± SD Median (IQR)	**5.75 ± 4.11** **5.0 (5.75)**	**9.24 ± 4.87** **10.0 (7.0)**	**0.031**
No diagnosis Doubtful diagnosis Clear diagnosis	8 (66%) 2 (16%) 2 (16%)	12 (35%) 8 (24%) 14 (41%)	0.155
Depression			
Mean ± SD Median (IQR)	**2.83 ± 3.54** **1.5 (5.0)**	**6.97 ± 4.79** **6.0 (7.5)**	**0.004**
No diagnosis Doubtful diagnosis Clear diagnosis	11 (92%) 0 (0%) 1 (8%)	23 (68%) 9 (26%) 2 (6%)	0.254

Results are shown as mean ± SD and median (IQR) of the indicated subscales. Mann–Whitney *U*-test and chi-square test were used for comparison. *P*-values <0.05 were considered statistically significant. Significant values are displayed in bold, while trends (*p* < 0.1) are in italic.

SD, standard deviation; IQR, interquartile range.

## Discussion

In our study we analyzed, to our knowledge, for the first time the allergological risk and the psychological structure of patients with vaccination hesitancy. We assessed patients seeking advice on COVID-19 vaccinations at our allergy center. Additionally, we examined regular patients with severe allergies and a history of anaphylactic reactions as a control group, considering the potential role of anxiety and depression. Both patient groups were similar in age, making the COV patient group representative of our regular patient population. However, the vast majority of patients presenting for COVID vaccination were female. Interestingly, female predominance was also seen in documented reactions after COVID vaccination (17) and other vaccines (5) and in a previous study regarding anxiety toward COVID vaccines in patients with a history of severe allergic reactions (7).

While all patients in our COV patient group perceived themselves as having an allergic risk during vaccination, only 86% had a known or confirmed allergy, and merely 28.6% reported a history of systemic allergic reactions. This was notably lower than the comparison group without vaccine hesitancy, where two-thirds had experienced anaphylaxis. This is in contrast to the literature where unvaccinated patients had a history of anaphylaxis more often (18). Furthermore, none of the COV patients had mastocytosis, a condition often associated with an increased risk of anaphylaxis, where close monitoring had been advised by allergy societies (19). However, no increased risk in this patient group could be verified in the course of vaccination programs and the pandemic (20).

The COV patients showed no variance in allergic comorbidities but displayed a higher prevalence of allergies across multiple categories (see **Figure 1**). They were notably more likely to have a known drug allergy to oral or injectable drugs compared to our control group. Consequently, we hypothesize that previous negative encounters with drugs contribute to vaccine hesitancy. Notably, patients with a history of drug allergy have been reported to be at a higher risk for urticaria following COVID vaccination (18). However, this was not reported back to us after the vaccinations.

In our clinic, the HADS was used in order to screen for anxiety and depression. Other scoresheets like the Patient Health Questionnaire-8 or -9 (PHQ-8 and PHQ-9) screening tool for depression (19) and the Generalized Anxiety Disorder Scale (GAD-7) to screen for anxiety (21) are more often recommended lately because they map onto the DSM5 and ICD-10 criteria. However, as a screening tool for routine assessments, the HADS is practicable, short, and easy and performs comparable to other tools (22). De Almeida Macêdo et al. (23) found no significant gender differences in HADS-A accuracy but noted that HADS-D might be less accurate for both genders (24). In our study, female patients had notably higher anxiety levels but had no discernible difference in depression scores. Generally, women are believed to experience depression about twice as often as men (25). Hence, it is unclear if the absence of gender disparity in depression is due to HADS-D’s limitations or the specifics of our patient cohort. Using the HADS, we found significantly higher rates of anxiety and depression in our COV patient group compared to those seeking routine allergological examination. With a female predominance in the COV group, we focused on female patients in both groups to rule out gender effects, still observing a similar trend. Similarly, when comparing only male patients, a trend toward higher depression levels was noted in the COV group, though anxiety differences were not confirmed. It is conceivable that concerns about COVID vaccination may contribute to elevated anxiety levels, although other authors (4, 26) could not verify a correlation between anxiety and vaccine hesitancy. We found no variance in anxiety levels among the patients in the COV group based on whether they had received a COVID vaccine (with a suspected reaction) or not. However, notably higher levels of depression were evident in patients without prior vaccination, yet there was no correlation between specific vaccines received and depressive symptoms.

Overall, in all our patients, anxiety was strongly and significantly correlated with depression. Anxiety and depression showed a weak positive correlation with total IgE serum levels, but not with any of the other analyzed clinical or lab parameters. A correlation between anxiety and depression has also been seen in patients with chronic urticaria. However, in this study, higher total IgE serum levels were not observed in patients with chronic urticaria and depression (27).

Our data raises the question why some patients, without a specifically higher risk of allergies, presented with vaccination hesitancy. A meta-analysis showed correlation with female sex, being 50 years old or younger, single, unemployed, education, and considering COVID-19 vaccines as unsafe in association with a higher risk of vaccination hesitancy (28). Most of these elements had not been assessed in our study, and we cannot say if, besides female predominance, some of these factors would have been present in our patients.

Though roughly a third of the patients had medical histories with mostly unclear reactions to COVID vaccination, prick testing with vaccine adjuvants yielded no positive reactions in the tested patients. Some patients declined testing due to fear of severe reactions or missing their appointments. Positive reactions in such tests are rare and often of limited significance (10). Since negative results do not entirely rule out allergic reactions, we offered vaccination under allergological supervision, with half of the patients agreeing after testing and more rarely patients after consultation only. We speculate that the higher vaccination rate post-prick testing reflects a perceived sense of security. Notably, patients declining vaccination had higher anxiety and depression scores. Overall, no patient had allergological contraindications for COVID vaccination according to the assessments.

In general, rates of self-reported allergic reactions after COVID vaccination were higher in patients with self-reported high-risk allergy history (17). Therefore, allergists play an important role in identifying those patients who actually have an elevated risk. Then, it is recommended that those patients with allergic risk are vaccinated by specialists (7). In our study, all patients with an alleged elevated risk tolerated vaccination at our center well without any immediate-type allergic reaction.

Limitations include the exploratory nature of our study with low patient numbers and a retrospective, single-center design. The use of the HADS questionnaire as a self-report tool may limit clinical relevance, and it does not cover the entire spectrum of anxiety and depression. Additionally, our study mainly included middle-aged adults, potentially affecting the generalizability to other age groups like children. Anamnestic data on allergies and comorbidities were largely self-reported. The final outcome of COVID vaccinations for some patients remains unknown, as they may have been vaccinated elsewhere or had unreported reactions later on. Nevertheless, our study underscores the importance of addressing psychological factors in allergological patients with vaccination hesitancy, a topic of recurring significance across different vaccine types.

In conclusion, vaccination hesitancy could be frequently elicited by negative experiences with drugs and possible drug allergies, particularly among female patients who also showed significantly elevated anxiety and depression scores. Prick testing resulted in a high vaccination rate, while discussions and personalized advice without testing rarely led to vaccinations. We think that this study shows that allergists should have an important role in future vaccination programs. Ideally, a multi-professional team including psychological care besides allergologists may be able to help provide reassurance and improve the vaccine hesitancy in patients with allergological background.

## Data availability statement

The original contributions presented in the study are included in the article/**Supplementary Material**. Further inquiries can be directed to the corresponding author.

## Ethics statement

The studies involving humans were approved by Johannes Kepler University Ethic Commission. The studies were conducted in accordance with the local legislation and institutional requirements. Written informed consent for participation was not required from the participants or the participants’ legal guardians/next of kin in accordance with the national legislation and institutional requirements.

## Author contributions

NK: Data curation, Formal analysis, Investigation, Writing – original draft. VP: Writing – review & editing, Investigation. WH: Resources, Supervision, Writing – review & editing. SA: Supervision, Writing – review & editing, Conceptualization, Data curation, Project administration.
