# Supplementary material for: Vaccine hesitancy in patients presenting to a specialized allergy center: clinical relevant sensitizations, impact on mental health and vaccination rates
Source: Front Immunol. 2024 May 17;15:1324987. doi: 10.3389/fimmu.2024.1324987 (PMC11140087; doi:10.3389/fimmu.2024.1324987)
Supplement: Supplementary file 1 [file DataSheet_1.docx]

**Supplements**

***Suppl. figure 1***

Suppl. figure 1: Prior anaphylaxis in Group ALL and COV. Number of patients are given per patient group. The colored (green, orange) parts represent the patients with prior anaphylaxis, the grey parts the patients without prior anaphylaxis grade ≥ 2.

***Suppl. figure 2a +b***

***
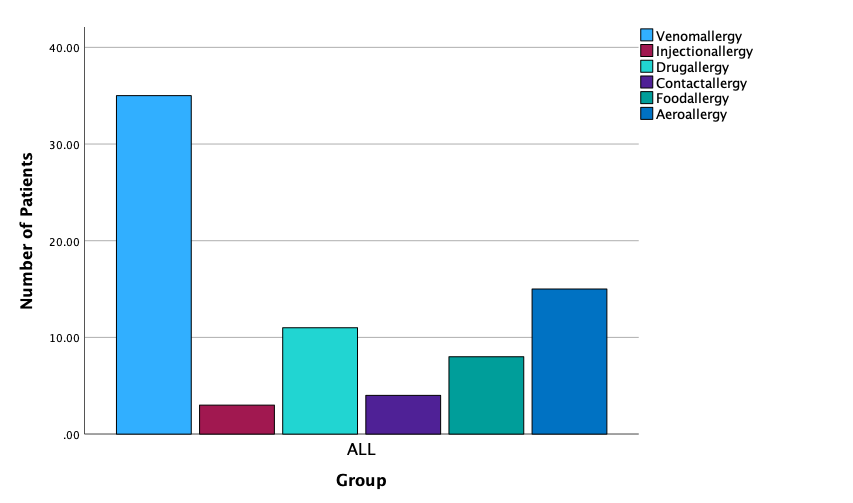
***

***
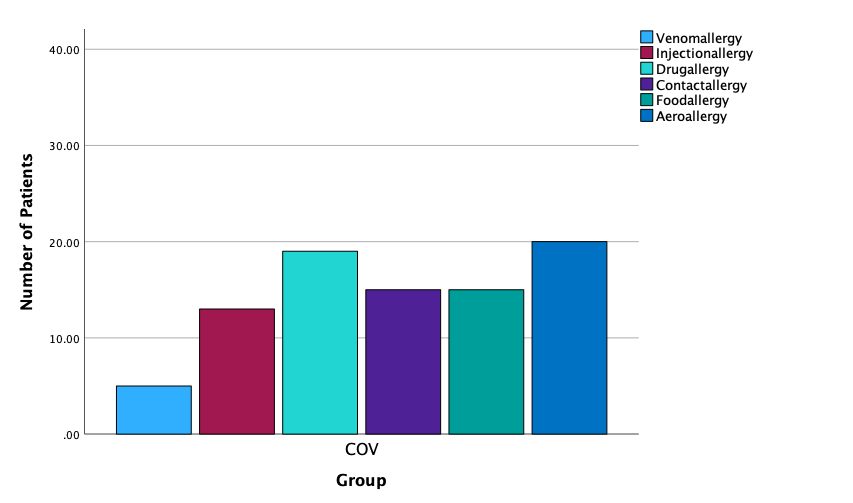
***

Suppl. figure 2: Different categories of allergies in patient group ALL (A) and COV (B). Prevalence in absolute numbers per category is depicted.

***Suppl. figure 3a + b***

***
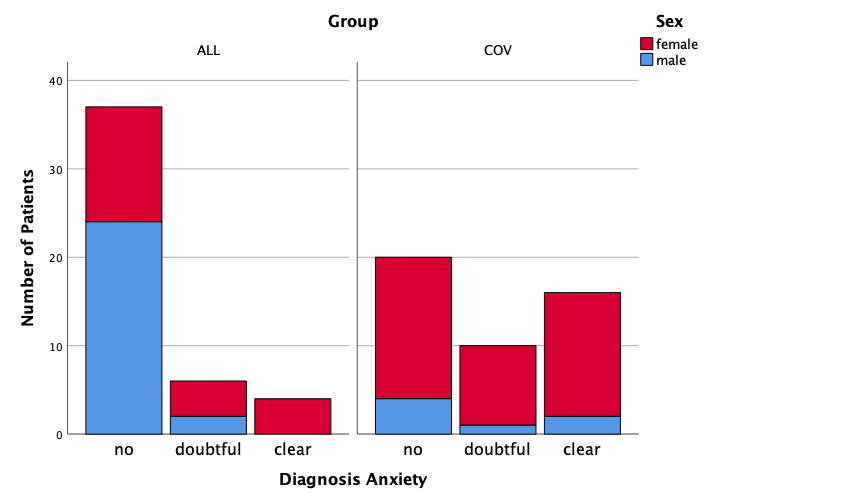
***

p=0.001

***
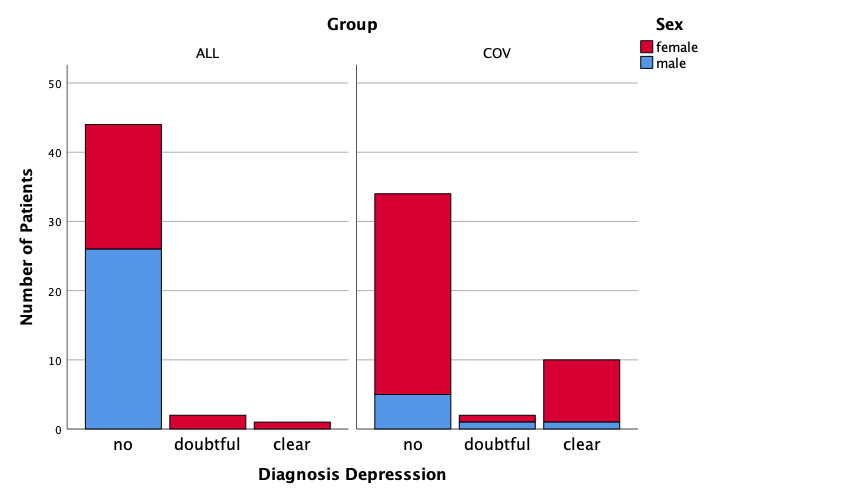
***

p=0.013

Suppl. Figure 3: Diagnosis for anxiety in groups ALL and COV following HADS-A (A) and –D (B). No diagnosed anxiety or depression (0-7 points), doubtful anxiety or depression (8-10 points) and clearly diagnosed anxiety or depression (>10 points). Absolute numbers of patients per diagnose group are depicted. Female and male patients are represented in different colors. Chi-Square-test was used for analyzing independence. P-values <0.005 were considered as statistically significant.

***Suppl. table 1***

|  | Patient  group ALL  n=47 | Patient  group COV  n=37 | Significance *p* |
| --- | --- | --- | --- |
| **Venom**  **allergy** | **35 (74.5%)** | **5 (13.5%)** | **<0.001** |
| **Allergy against injectable drugs** | **3 (6.4%)** | **13 (35.1%)** | **<0.001** |
| **Allergy against**  **oral medication** | **11 (23.4%)** | **19 (51.4%)** | **0.008** |
| **Contact**  **allergy** | **4 (8.5%)** | **15 (40.5%)** | **<0.001** |
| **Food**  **allergy** | **8 (17.0%)** | **15 (40.5%)** | **0.016** |
| **Aero**  **allergy** | **15 (31.9%)** | **20 (54.1%)** | **0.041** |

Table 4: Prevalence of different categories of allergies in patient cohort ALL and COV. The figures show the absolute frequency, numbers in brackets show the corresponding percentages. Chi-square test was used for comparison. P-values < 0.05 were considered as statistically significant. Significant values are displayed in bold.

***Suppl. table 2***

|  | **Male patients**  **n=33** | **Female patients**  **n=60** | **Significance p** |
| --- | --- | --- | --- |
| **Anxiety**  Mean ± SD  Median (IQR) | **4.5 ± 3.6**  **3.0 (5.0)** | **7.6 ± 4.7**  **8.0 (7.75)** | **0.001** |
| **Depression**  Mean ± SD  Median (IQR) | 3.5 ± 3.3  3.0 (4.5) | 5.1 ± 4.4  4.5 (5.0) | 0.101 |

Suppl. table 2: HADS score of all male and female patients. Results are shown as mean ± SD and median (IQR) of the indicated subscales. Abbreviations: SD – standard deviation, IQR – interquartile range. Mann-Whitney-U test was used for comparison. P-values < 0.05 were considered as statistically significant. Significant values are displayed in bold, trends (p <0.1) in italic.

***Suppl. table 3a + b***

|  | **Male patients of patient group ALL**  **n=26** | **Female patients of patient group ALL**  **n=21** | **Significance p** |
| --- | --- | --- | --- |
| **Anxiety**  Mean ± SD  Median (IQR) | **3.6 (± 2.6)**  **3.0 (4.25)** | **6.0 (± 4.1)**  **6.0 (6.5)** | **0.038** |
| **Depression**  Mean ± SD  Median (IQR) | 2.7 (± 2.3)  2.0 (4.25) | 3.6 (± 3.0)  3.0 (4.25) | 0.399 |

|  | **Male patients of**  **patient group COV**  **n=7** | **Female patients of patient group COV**  **n=39** | **Significance *p*** |
| --- | --- | --- | --- |
| **Anxiety**  Mean ± SD  Median (IQR) | 7.6 (± 5.3)  7.0 (11.0) | 8.5 (± 4.9)  8.0 (7.0) | 0.697 |
| **Depression**  Mean ± SD  Median (IQR) | 6.1 (± 5.0)  6.0 (8.0) | 5.8 (± 4.8)  5.0 (6.0) | 0.811 |

Suppl. table 3: HADS score of male and female patients in group ALL (A) and COV (B): Results are shown as mean ± SD and median (IQR) of the indicated subscales. Abbreviations: SD – standard deviation, IQR – interquartile range. Mann-Whitney-U test was used for comparison. P-values < 0.05 were considered as statistically significant. Significant values are displayed in bold, trends (p <0.1) in italic.

***Suppl. table 4***

|  | **Male patients of patient group ALL**  **n=26** | **Male patients of**  **patient group COV**  **n=7** | **Significance p** |
| --- | --- | --- | --- |
| **Anxiety**  Mean ± SD  Median (IQR) | 3.6 (± 2.6)  3.0 (4.25) | 7.6 (± 5.3)  7.0 (11.0) | 0.109 |
| **Depression**  Mean ± SD  Median (IQR) | *2.7 (± 2.3)*  *2.0 (4.25)* | *6.1 (± 5.0)*  *6.0 (8.0)* | *0.074* |

Suppl. table 4: HADS score of male patients in group ALL and COV: Results are shown as mean ± SD and median (IQR) of the indicated subscales. Abbreviations: SD – standard deviation, IQR – interquartile range. Mann-Whitney-U test was used for comparison. P-values < 0.05 were considered as statistically significant. Significant values are displayed in bold, trends (p <0.1) in italic.

***Suppl. table 5***

|  | **Patients with prior vaccination**  **n=11** | **Patients without prior vaccination**  **n=35** | **Significance p** |
| --- | --- | --- | --- |
| **Anxiety**  Mean ± SD  Median (IQR) | 6.4 ± 4.1  7.0 (7.0) | 8.9 ± 5.0  10.0 (7.0) | 0.124 |
| **Depression**  Mean ± SD  Median (IQR) | **2.3 ± 2.6**  **1.0 (5.0)** | **7.0 ± 4.8**  **6.0 (7.0)** | **0.002** |

Suppl. table 5: HADS score of patients with prior vaccination and without prior vaccination of group COV: Results are shown as mean ± SD and median (IQR) of the indicated subscales. Abbreviations: SD – standard deviation, IQR – interquartile range. Mann-Whitney-U test was used for comparison. P-values < 0.05 were considered as statistically significant. Significant values are displayed in bold, trends (p <0.1) in italic.
